# Graduate certificate on risk analysis for the Agrifood sector at the University of Buenos Aires

**DOI:** 10.3389/fbioe.2024.1378538

**Published:** 2024-02-26

**Authors:** Carmen Vicién, Clara Rubinstein

**Affiliations:** ^1^ School of Agriculture, University of Buenos Aires, Buenos Aires, Argentina; ^2^ ICCAS (Institute for Scientific Cooperation on Health and the Environment), Buenos Aires, Argentina

**Keywords:** risk analysis, graduate course, continuing education, Agrifood, biosafety, regulatory science, University of Buenos Aires

## Abstract

Argentina has an extensive experience in the biosafety assessment of transgenic crops. The regulatory framework celebrated 30 years of existence in 2021 and has pioneered the establishment of the biosafety systems in Latin America. During this period, Argentina’s regulatory framework evolved to keep up with the advancements in plant and animal biotechnology and in risk assessment criteria, as new knowledge and experience was being gained. However, despite the country’s agricultural tradition and experience in the adoption of innovations by the productive sector, dedicated, formal academic offerings training is lacking and this is also true for most countries in the world. Responding to this perceived need and going beyond biotechnology to include other regulated inputs used along the food production chain (chemicals, biologics, food additives, etc.), we developed a program to introduce graduates from diverse disciplines to the principles and practice of Risk Analysis (Assessment, Management and Communication) with focus on the Agrifood sector. In 2020, the School for Graduate Students of the School of Agriculture—University of Buenos Aires, approved two Certificates on Risk Analysis for the Agrifood Sector: *Conceptual Bases of Risk Analysis* and *Methodological Tools.* The first edition of the certificates was completed in December 2022 and the second one is presently ongoing. The fundaments, rationale, structure and objectives of these certificates are presented.

## 1 Introduction

The production, distribution and consumption of food involve different actors and is a strongly regulated sector. Crop protection products-including biologicals-, transgenic seed, food additives and adjuvants for the food industry, are the main inputs subject to regulatory oversight and therefore need to go through authorization processes before entering the market.

Biosafety risk assessment is focused on the potential impacts that these products or technologies might have on health or the environmental, or both. The risk assessment process is the technical analytical stage of risk analysis that regulatory agencies carry out. In general, this process is based on scientific criteria, country policies and analytical methodologies that are internationally accepted (OECD, 2005), but fundamentally, is a way of thinking and approaching problem resolution.

In many countries, there is a need for professionals trained and experienced in these methodologies in the fields of food safety, crop protection products (including biologicals) or transgenic organisms. Academic programs like the one described here provide a formal context to develop capacities in both public and private organizations. Career opportunities for professionals with these capacities are diverse, as regulatory affairs specialists for private sector developers in the agricultural, chemical or biotechnology fields, as risk assessors in the public sector, and also in academia.

In a risk averse society, where concerns about food and environmental safety are part of our everyday lives, the role of regulatory agencies is central and professional risk assessors that are skilled and can clearly communicate, are key to develop fit for purpose, solid risk-based regulations that build credibility and trust (OECD, 2021).

The availability of an educational space dedicated to fulfill this need, in Spanish, was considered a valuable contribution to our region, that would complement the formation of professionals in the different disciplines related to risk analysis for the agrifood sector.

The School of Agriculture of the University of Buenos Aires hosts the School for Graduate Students “Agronomy Eng. Alberto Soriano.” Along its 36 years of existence, the school formed over 1,000 professionals and currently offers over 100 Graduate Programs (Masters, PhDs, Specializations, Continuing Education courses and others) in different disciplines related to the Ag Sciences field, from Genetics and Crop Physiology, to Economy, Agribusiness and Natural Resources. Back in 2020, the School added two new correlative Certificates on Risk Analysis for the Agrifood Sector to their academic offerings (EPG -FAUBA).

These Certificates intend to introduce professionals to the general principles of Risk Analysis, familiarizing them with the analytical thinking process required to identify risks and address their management, under the Regulatory Science framework. These competences are key to identify, estimate or quantify risks and define acceptable levels, as well as deal with the management or mitigation measures that might be necessary.

But beyond strictly technical skills, another equally important set of competences involves other abilities like risk communication, consensus building and diplomacy, as risk assessment and regulatory decision making involve not only conducting a technical analysis but also informing risk managers, policy makers and, increasingly, responding to societal demands.

As mentioned, regulatory science is at the foundation of this course, as a scientific discipline on its own, which proposes plausible risk hypotheses that result from the Problem Formulation exercise (Wolt et al., 2010), and tests them applying appropriate methodologies and also generating innovative methods and standards.

Risk assessment it’s a multidisciplinary endeavor that requires experts from different fields (chemists, biologists, geneticists and toxicologists, medical doctors, agronomists, microbiologists and animal scientists) and is a scientific procedure based on regulatory science (Deluyker, 2017).

## 2 Why two parts? topic areas and curriculum

The first, introductory part “Conceptual Bases,” gives an overview of risk assessment principles and approaches (like problem formulation and the identification of risk hypotheses) and the scientific rationale used in their development.

The first module provides a detailed overview of the surrounding context (productive, economic, societal perception, regulatory). The second module, focuses on the different disciplines that are key in the generation of experimental evidence and its interpretation for risk characterization: Toxicology, Food Technology, Biotechnology, Regulatory Toxicology, Epidemiology, Food Composition and Nutrition and Statistics are part of the curriculum. Although this first part is more theoretical and informative, examples and cases are discussed and practical situations are presented to the students for their debate and resolution.

The second part, “Methodological Tools,” focuses on the practice of Risk Analysis and the important factors to consider when going through the process, from the risk characterization stage and the decision-making process, to the normative aspects. Given Argentina’s experience on the subject, regulation of gene editing has been included as a topic, as well as the regulatory approach for agricultural biologicals.

As mentioned above, this part includes contents that have not traditionally or formally been part of risk assessment trainings but that, in our view and experience, are very important aspects to consider. An example of this is the Communication module, discussing the importance of adequately communicating decisions and management considerations, if any, to different stakeholders: risk managers, end users and society at large.

**FIGURE 1:**
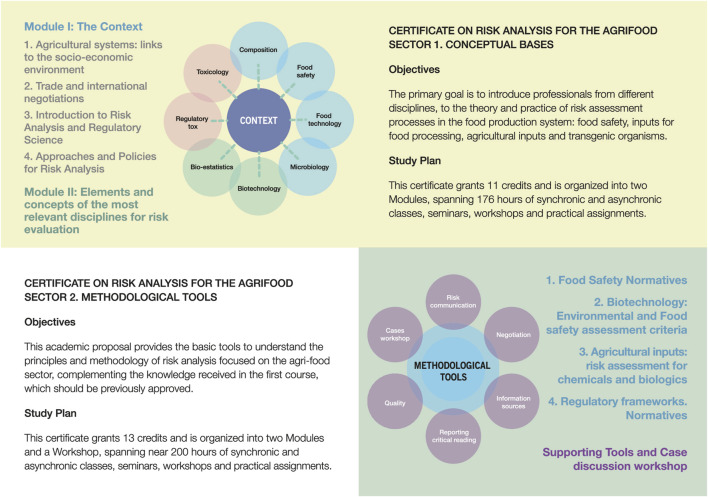
General Structure of the Graduate Certificates. Part I and II are correlative and grant credits that can be accepted by other Graduate Programs of the School.

Both parts have considerable time dedicated to lectures, invited speakers, discussion with experts and group work, as one of the objectives of these courses is to foster interactions and consensus building, as it happens in real life. A general scheme of the Certificates is summarized in Figure 1.

## 3 Faculty, students’ profile and first experience

These certificates include over 20 lecturers, all of them specialists in their fields, plus invited experts that share experiences, participate in discussions with the students, or speak about specific topics of interest. Agronomists, molecular biologists, breeders, biochemists, toxicologists and food technologists teach “Conceptual Bases.” Additionally, experts in topics like Communications, Quality systems, Research Integrity, Ecotoxicology and Regulations, teach “Methodological Tools.”

Our Faculty comes from different sectors, government regulatory agencies, private companies or academia, bringing a high level of expertise and diversity to the courses, as our intention is to give students the opportunity to learn about the concepts and the practice of risk analysis first hand from the experts, and understand how the processes work under real life conditions, as far as possible.

Classes are 3 h long, once a week, with some additional days along the year dedicated to group examinations and complementary lectures or case discussions.

The certificates are open to university graduates from diverse disciplines (Agronomy, Biology, Chemistry, Biochemistry, Genetics, Medicine, Animal Science, Biotechnology, Microbiology, Toxicology, etc.), and the online modality allows to have students from different Spanish speaking countries.

The first cohort was composed of 15 experienced professionals in the fields of Agronomy, Toxicology, Biology, Biotechnology and Biochemistry, including two regulators. This group came from both the private and public sectors, from different states in Argentina, and also from Paraguay. Eleven students completed both certificates.

After each module, surveys were conducted among students to gather opinions and use the feedback to introduce improvements. Results have been very positive so far, highlighting the quality of the contents and lecturers and the usefulness and applicability of the courses to their professional practices.

The second cohort completed the first certificate in 2023 and will continue to take the second part in 2024. After our first experience, this second edition will introduce some modifications, redefine specific contents and include international invited speakers, which will give students additional perspectives.

Group discussions are a central part of both courses, as these promote inter-disciplinary exchange and consensus building, the two main pillars of risk assessment processes under real life conditions around the world. Diverse backgrounds are welcome and have not prevented performance or participation so far.

## 4 Discussion

Functional risk assessment bodies in the biosafety field require continued education and training efforts to form professional profiles. The lack of formal intra-agency processes that ensure continuity in this formative process, is a challenge that is not exclusive of a particular country or region, but is an extended problem.

Capacity building initiatives that have been and are being implemented are numerous and highly valuable experiences, in particular for developing countries, however, the lack of continuity, high staff rotation or insufficient funding, undermines these efforts.

As stated in the recent “Anticipatory regulation in an age of disruption” Nesta report: “ … regulation and regulatory practice need to be recognised as crucial elements of the industrial strategies that are being developed and implemented in the United Kingdom and elsewhere. Indeed, the quality of regulatory practice in relation to innovation will be an increasingly important source of competitive advantage in the global economy” (Armstrong et al., 2019).

In fact, science based regulatory frameworks are not static, on the contrary, this is a dynamic field that needs to evolve and adapt as new knowledge, innovation and societal demands increase (Vicién and Trigo, 2017). These demands (like the reduction in animal testing), create new requirements to adapt criteria and methodologies, develop new methods and predictive tests. In turn, this means continuing education and updates to regulators and risk assessors is and will continue to be a permanent need.

Academic offers that provide access to high quality, updated formation and to experts in the different fields, will contribute to develop capacities for risk assessment. In our case, certificates are open to graduates interested in this field, that will incorporate problem solving tools and criteria applicable to the private or public sectors.

Our first experience with the graduate certificates in Risk Analysis for the Agrifood sector is the first of its kind in the region and is held in Spanish. Both certificates provide tools and criteria to introduce students to the principles and practice of risk analysis, from problem formulation to risk characterization and management, including risk perception and communication considerations.

Professional, transparent and efficient regulatory systems, applying state of the art criteria and methodologies, have the highest credibility and build societal trust. This is true at the global level, but specially so in developing countries, some of which still find barriers for innovations to reach farmers and consumers. This is particularly true for local innovations by public institutions or small innovative companies, and one reason is the lack of highly trained assessors. Regulatory systems that are up-to-date, fit for purpose and adaptable to current and future innovations will be increasingly needed in order to bring safe, beneficial products and technologies to consumers.

Going forward, we plan to turn both certificates into a Master’s Degree, compiling both parts into one and adding a final work (Master’s thesis). Students from previous editions, will be able to use their credits to obtain this degree.

We are in contact with other institutions beyond Argentina, with similar approaches to higher education in the Risk Analysis field, and look forward to establishing agreements to collaborate and exchange students and faculty in the near future.

## Data Availability

The original contributions presented in the study are included in the article/Supplementary material, further inquiries can be directed to the corresponding author.
